# Aboveground biomass in seven tropical forest patches of Western-Africa: comparison of manual inventory and terrestrial laser scanning

**DOI:** 10.1186/s13595-026-01329-7

**Published:** 2026-04-24

**Authors:** Samuel Hepner, Georges Alex Agonvonon, Daniel Kükenbrink, Chima Iheaturu, Fortuné Azihou, Brice Sinsin, Chinwe Ifejika Speranza

**Affiliations:** 1 Land Systems and Sustainable Land Management, Institute of Geography, University of Bern, Bern, Switzerland; 2 Remote Sensing, Swiss Federal Institute for Forest, Snow and Landscape Research, Birmensdorf, Switzerland; 3 Laboratory of Applied Ecology, Faculty of Agricultural Sciences, Université d’Abomey-Calavi, Cotonou, Benin

**Keywords:** Allometric equation, Automatic point-cloud segmentation, Edge effects, Forest fragmentation, Tree species richness, Wood density

## Abstract

**Key message:**

Aboveground biomass (AGB) increased from forest edge to forest interior in small forest patches of Western-Africa. In plots of 0.25 ha, AGB did not correlate with tree species richness or wood density. AGB in these unprotected forest patches was lower than in the nearby protected forests. AGB obtained from manual inventory and terrestrial laser scanning correlated moderately.

**Context:**

In Western-Africa, small, unprotected forest patches amidst agricultural lands provide vital ecosystem services like carbon storage. However, accurately measuring aboveground biomass remains challenging, and terrestrial laser scanning (TLS) might become an accurate, non-destructive method.

**Aims:**

This study explored AGB, its spatial distribution, and relationships with ecological determinants, and compared AGB estimated from manual inventory with that from TLS.

**Methods:**

We established 109 plots and inventoried 9591 trees across seven forests in Togo, Benin, Nigeria, and Cameroon. AGB was obtained from allometric equations using diameter and tree heights, as well as from segmented point-clouds. Plot-level AGB was extrapolated to the entire forest.

**Results:**

AGB in forest patches ranged from 85 to 259 Mg/ha, which is lower than in nearby protected forests. Forests close to the equator have generally higher AGB, and most forests showed reduced AGB and wood density close to forest edges. AGB showed no correlation with wood density, structural complexity, and tree species richness. AGB estimations by manual inventory and TLS correlated moderately.

**Conclusion:**

Our findings highlight the value of ground-based methods and the need to connect and protect forests as carbon reservoirs.

## Introduction

### Forest loss, fragmentation, and persisting patches in Western-Africa

Tropical forests are being cleared globally at alarming rates (Schelhas and Greenberg 1996; Hansen et al. 2013; Poorter et al. 2021). In Western-Africa, more than 80% of the 1900 forest extent has been lost, mainly due to the growing human population clearing forests for agriculture (Aleman et al. 2017; Curtis et al. 2018; Amani et al. 2021; Akinyemi and Ifejika Speranza 2022). In addition to deforestation, large contiguous forests have been fragmented into numerous small patches (Taubert et al. 2018; Traoré et al. 2024; Wingate et al. 2022). Between 2000 and 2010, the number of forest fragments increased by 42% in Africa (Fischer et al. 2021). Fragmented areas are particularly affected by forest loss (Dangbo et al. 2020), and remaining forest patches are vulnerable to edge effects, such as fire, desiccation, and altered species composition (Hill and Curran 2003; Ibáñez et al. 2014; Laurance 2004; Taubert et al. 2018).

Despite this deforestation trend, thousands of small forest patches (0.5—1000 ha) persist in isolation across the Western-African landscape, and in Togo, Benin, Nigeria, and Cameroon alone, over 400,000 patches have been detected in the Guineo-Congolian forest and the Guinea Savanna zones (Wingate et al. 2023). These patches are characterized by trees exceeding 5 m in height and a canopy cover greater than 30% (Food and Agriculture Organization of the United Nations (FAO) 2016; Wingate et al. 2022). They are crucial for biodiversity conservation and climate regulation, including carbon storage and sequestration (Lewis et al. 2015).

Previous research on these forest patches assessed structural complexity and its degradation under edge effects (Hepner et al. 2025a), documenting reductions in stand structural complexity, basal area, and tree height near forest edges and in disturbed patches. Other studies documented tree species diversity across different disturbance and bioregion types (Agonvonon et al. 2026) and explored local perceptions of forest degradation and use (Hepner et al. 2025c). Building on these findings, the present study investigates aboveground biomass (AGB), its spatial distribution, and comparisons between manual inventory and terrestrial laser scanning. Together, these studies provide an integrated understanding of forest condition, carbon storage, biodiversity, and socio-ecological dynamics in small, unprotected forest patches of Western-Africa.

### Aboveground biomass (AGB)

Tropical forests store most of the terrestrial aboveground carbon and are central to climate mitigation and biodiversity conservation (Chave et al. 2019; Ameray et al. 2021). In this context, aboveground biomass (AGB), largely contained in woody compartments of trees (Williams et al. 2013), is a key parameter for assessing global carbon balance, timber management, and ecosystem services. International initiatives such as REDD + (Reducing Emissions from Deforestation and forest Degradation and the role of conservation, sustainable management of forests and enhancement of forest carbon stocks in developing countries), the Kunming-Montreal Global Biodiversity Framework, and other schemes require robust estimates of AGB to monitor commitments and guide management (Food and Agriculture Organization of the United Nations (FAO) and United Nations Environment Programme (UNEP) 2020; Convention on Biological Diversity (CBD) 2021; International Union for Conservation of Nature (IUCN) 2022; Turia et al. 2022).

In Western-Africa, however, most AGB studies have concentrated on large, formally protected forest blocks or a few commercially important species (Basuki et al. 2009; Chenge and Osho 2018; Aabeyir et al. 2020; Atsri et al. 2020; Arouna et al. 2021). Small and unprotected forest patches remain underrepresented, despite their abundance and ecological importance (Wingate et al. 2023). These patches are particularly exposed to edge effects, including higher tree mortality, windthrow, and fire, which can substantially reduce biomass (Laurance et al. 1997, 2000; Ordway and Asner 2020; Giancola et al. 2024). Structural changes, such as reduced tree height for a given diameter (Nunes et al. 2023), and the loss of large animal seed dispersers (Lewis et al. 2015), further contribute to lower AGB compared to continuous forests.

Understanding AGB in small forest patches is therefore crucial. They may serve as analogues of the future landscape if fragmentation continues (Tabarelli et al. 2008; Taubert et al. 2018), and their carbon dynamics will determine whether they act as persistent carbon sinks or sources. Yet, temporal trends of AGB in these fragments remain poorly understood and contested (Wingate et al. 2023). In addition, little is known about how the biomass of these small forest patches compares with that of other nearby forests in the region, whether larger, formally protected, or similar in size and management, although this contrast is central for evaluating their role in regional carbon budgets.

### Challenges of quantifying aboveground biomass

Quantifying AGB in tropical forests is notoriously difficult due to high species richness, variable tree allometries, and the presence of very large individuals (Hemp et al. 2017; Cazzolla Gatti et al. 2022; Calders et al. 2022a, b). While destructive harvesting remains the most accurate method (Ketterings et al. 2001), it is rarely feasible, and indirect approaches such as manual inventories and remote sensing are commonly used (Clark and Kellner 2012). However, inventorying even a single hectare of tropical forest is logistically demanding and expensive (Chave et al. 2019; ForestPlots.net et al. 2021), and such efforts remain scarce in Western-Africa (Harris et al. 2021).

Satellite-derived biomass maps provide valuable regional and global coverage, but their accuracy is limited by the paucity of representative ground data and by strong structural heterogeneity in Afrotropical forests (Chave et al. 2019; Araza et al. 2022). These limitations are particularly acute in small forest patches, which are often excluded from large-scale inventories and misclassified by coarse-resolution remote sensing products. As a result, current maps show discrepancies of more than 150 Mg ha⁻^1^ in Western-Africa (Araza et al. 2022), and the biomass of small patches remains largely unvalidated.

Emerging technologies such as terrestrial laser scanning (TLS) offer a promising complement to manual inventories. TLS captures forest structure in three dimensions, providing accurate estimates of tree size and canopy height without destructive sampling (Calders et al. 2020; Terryn et al. 2024). While TLS has been tested in temperate and Amazonian forests, its application in Western-Africa is minimal and absent from Togo, Benin, and Nigeria (Momo et al. 2018, 2020). Forest patches are a particularly relevant test case, as they combine high floristic diversity, structural heterogeneity, and strong edge effects within small areas, posing both logistical challenges and opportunities for TLS validation. Moreover, comparing the AGB of these patches with other regional forests can clarify whether small remnants store carbon proportionally or show systematic differences across the landscape.

To shed light on aboveground biomass and its quantification in these understudied ecosystems, we addressed the following questions:

1. What is the current AGB and carbon storage in the studied forest patches, and how is it spatially distributed?
  H: We expect that the amounts and spatial patterns of AGB and carbon vary across the forest patches, indicating environmental and disturbance gradients.
2. Which forest characteristics correlate most with AGB?
  H: We expect basal area, tree height, and wood density to correlate most with AGB.
3. How does the AGB of these patches compare with that of other forests in the region?
  H: We expect to find lower AGB in isolated forest patches as compared to larger forest areas, due to edge effects.
4. How does the AGB estimated from the manual inventory compare to the AGB obtained by TLS?
  H: We expect that AGB obtained by TLS will show a positive correlation with AGB derived from manual inventories across forest patches.

To address these research questions, we focused on seven forest patches in Togo, Benin, Nigeria, and Cameroon, spanning diverse forest types and ecological conditions.

### Study area

**Fig. 1:**
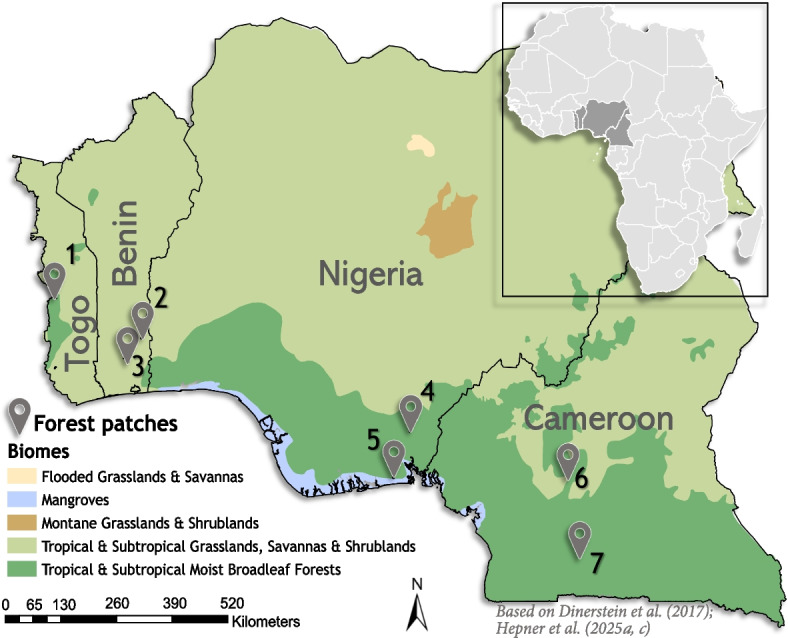
Seven forest patches were selected in Western-Africa, spanning the Tropical & Subtropical Grasslands, Savannas & Shrublands (light green) and the Tropical & Subtropical Moist Broadleaf Forests (dark green) of Togo, Benin, Nigeria, and Cameroon. Forests are numbered as follows: 1. Koui, 2. Ewè-Adakplamè (also known as Kouvizoun sacred forest Adakplamè-Ewè), 3. Hlanzoun (also known as Lokoli), 4. Iko, 5. Ikot, 6. Mbangassina, 7. Ngam-Kondomeyos

These patches represent both Tropical and Subtropical Grasslands, Savannas, Shrublands, and Moist Broadleaf forests (Fig. 1; Dinerstein et al. 2017; see Appendix Table 4 for details). These remnants include semi-deciduous forests (Koui and Ewè-Adakplamè; also known as Kouvizoun sacred forest Adakplamè-Ewè), soil-mediated swamp forests (Hlanzoun, also known as Lokoli, and Ikot), and moist forests (Iko, Mbangassina, and Ngam-Kondomeyos). Together, they capture a broad ecological gradient and taxonomic diversity, with frequent tree families such as Moraceae (e.g., *Treculia africana* Decne Ex Trécul), Fabaceae (e.g., *Gilbertiodendron dewevrei* (De Wild.) J.Léonard), and Myristicaceae (e.g., *Pycnanthus angolensis* (Welw.) Warb.).

Despite lacking formal protection, these forest patches have persisted since at least the 1970 s, surrounded today by croplands, agroforestry systems, and wetlands (Hansen et al. 2013; Wingate et al. 2022). They span pronounced environmental gradients: mean annual precipitation ranges from 1000 to 1300 mm in Koui, Ewè-Adakplamè, and Hlanzoun, and from 1500 to 3000 mm in Iko, Ikot, Mbangassina, and Ngam-Kondomeyos; mean annual temperatures lie between 23 and 28° C (Hijmans et al. 2005). Elevations extend from 15 m above sea level in Ikot to 700 m in Ngam-Kondomeyos (Jarvis et al. 2008). Soil types predominantly include Acrisols, Lixisols, and Ferralsols, with Gleysols and Fluvisols occurring in the swamp forests (International Union of Soil Sciences (IUSS) Working Group 2015). Including such ecologically diverse sites strengthens the spatial coverage of AGB assessments across Western-Africa (Lewis and Pickavance 2024) and provides essential ground-truthing data for satellite-based forest archetypes (Wingate et al. 2023).

## Material and methods

### Data collection

Between September 2022 and March 2023, we installed 109, 50 × 50 m plots across seven forest patches, following plot size recommendations by Chave et al. (2004) and Duncanson et al. (2021). Within each patch, plots were distributed randomly, constrained by a minimum inter-plot distance of 50 m, accessibility, and human security considerations. This spacing prevents plot overlap and follows common practice in tropical forest inventories (e.g., Hepner et al. 2025a). While some spatial autocorrelation may remain, our analyses focus on stand-level comparisons and observed relationships rather than spatial prediction or model validation, where spatial autocorrelation poses stronger methodological challenges (e.g., Ploton et al. 2020a, b). To ensure representativeness, plots were located in internally homogeneous areas (avoiding canopy gaps or abrupt changes in vegetation structure, composition, and topography). Depending on forest patch size (20–1160 ha), we installed 6–21 plots per forest to ensure representative coverage.

The manual forest inventory included all trees with a diameter at breast height (DBH) ≥ 10 cm. Smaller trees, dead logs, lianas, and palms were disregarded since they contribute little to AGB (Ali et al. 2019c; Atsri et al. 2020; Duncanson et al. 2021). The position of each tree was taken with a handheld GPS (Garmin GPSMAP 66i). While occasional device readings suggested a precision of around ± 3 m, actual horizontal accuracy likely varied with canopy density, generally falling within the 5–10 m range (Garmin Ltd. 2025). DBH was measured with a diameter tape (0.1 cm precision), individual tree height was estimated with a clinometer, and tree species were identified with local botanists and later confirmed in national herbaria.

**Fig. 2:**
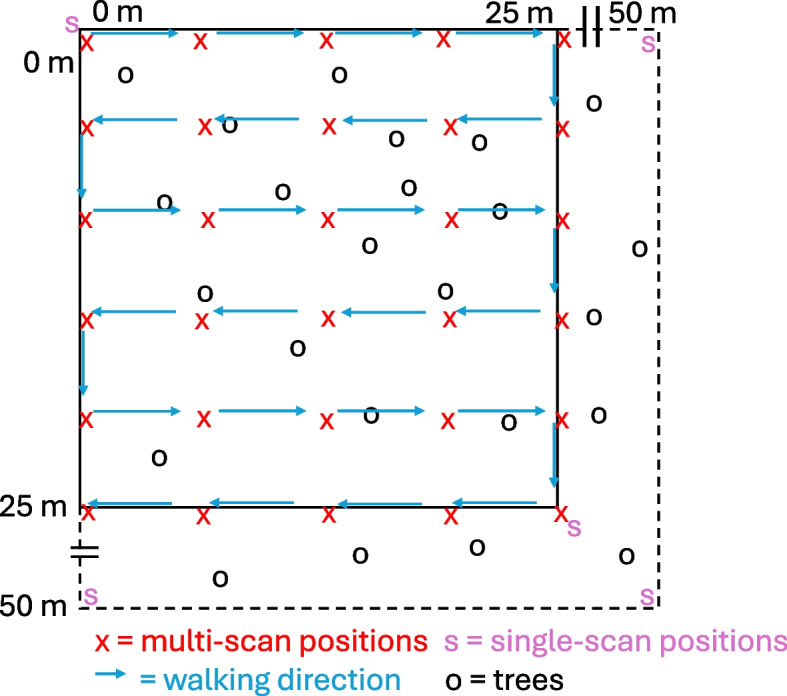
Scan sampling strategy in 50 × 50 m plots with 25 × 25 m subplots. Distances and number of trees are not to scale

We established a 25 × 25 m subplot in a random corner of 86 plots (see subplot level 1 in Food and Agriculture Organization of the United Nations (FAO), 2004). This approach facilitated orientation in the dense forests, as two subplot sides coincided with measuring tapes from the main plot, and the four subplot corners were already marked with colored poles. Within each subplot, all trees were tagged with unique numbers and registration markers (FARO Technologies Inc. 2017). We scanned the subplot with a terrestrial laser scanner (FARO M70) with 24.2 MPts, 0.044°/pt, and color mode, which took ca. 4.5 min/scan. We followed a continuous chain, always scanning the markers twice to allow subsequent co-registration (Wilkes et al. 2017; Martin-Ducup et al. 2021; Tao et al. 2021). Depending on forest density, we conducted approximately 30 scans per subplot, with scan positions spaced around 6 m apart (Fig. 2). Additionally, we took five single scans in the corner and the center of the plots to quantify the stand structural complexity index (SSCI, Ehbrecht et al. 2017; Hepner et al. 2025a). Scans were only taken when there was no rain, no wind, and no moving people close to the scanner.

### Data analysis

#### Manual inventory data

In total, 9,591 individual trees of 369 different species were identified. 281 trees (3%) of 25 genera (7%) could only be identified to the genus. To calculate AGB of these trees, the BIOMASS-package (Réjou-Méchain et al. 2017) was run (see also Mo et al. 2023; Ploton et al. 2020a, b) in R (version 2023.06.0, R Core Team 2023). Due to the potential inaccuracy of tree heights measured with a clinometer, we applied a local allometric model (log2, residual standard error (*RSE)* = *4.96 m*) to adjust the estimates (Réjou‐Méchain et al. 2017). Species-specific wood densities from the Global Wood Density Database (Zanne et al. 2009) were assigned to 5,062 trees (53%), genus-averaged densities to 3,011 trees (31%), and plot averages to 1,518 trees (16%). We applied the pantropical allometric equation by Chave et al. (2014), which is commonly used in tropical AGB studies (Cuni-Sanchez et al. 2021; Davies et al. 2021; Zemp et al. 2023 Eq. 1):

1 $${AGB}_{m}=0.0673*{\left(\rho {D}^{2}H\right)}^{0.976}$$

where AGB_m_ = aboveground biomass (kg) from manual inventory, $\rho$ = wood density (g/cm^3^), D = diameter at breast height (cm), and H = tree height (m).

Subsequently, a Monte Carlo test was applied to quantify error propagation and corresponding credibility at the plot level (2.5% and 97.5%, Appendix Fig. 6). AGB_m_ was converted to carbon by the factor 45.6% (± 0.2), which is the mean for tropical angiosperms (Martin et al. 2018). Species richness was standardized for differences in tree abundance among plots using individual-based rarefaction and extrapolation (Chao et al. 2014) implemented in the iNEXT R-package (Hsieh et al. 2024). Further, species richness was estimated from species–abundance data (stem counts per plot) with datatype = "abundance" and q = 0, providing rarefied, extrapolated, and asymptotic richness values with associated standard errors. To test the relationships between spatial and ecological determinants of aboveground biomass (AGB), including distance to the edge, basal area, tree height, wood density, tree species richness, number of trees, SSCI, and canopy openness, we applied linear mixed-effects models. This approach accounted for the random effects of individual forests as a single factor, using the lme4 and lmerTest R packages (Bates et al. 2015; Kuznetsova et al. 2017). We evaluated four model variations: fixed intercept and fixed slope, fixed intercept and varying slope, varying intercept and fixed slope, and varying intercept and varying slope. The model with the lowest Akaike information criterion (AIC) was retained (Bozdogan 1987), and model fit was assessed using Restricted Maximum Likelihood (REML). Log-transformations were applied to diameter and tree height to improve model fit, while wood density was modeled on the original scale.

#### Extrapolation from plot to forest

We estimated forest-wide AGB by extrapolating AGB_m_ from the 109 plots (50 × 50 m) using its relationship with canopy height. First, in QGIS (version 3.28.7-Firenze, QGIS Development Team 2023), the minimum bounding geometry function was applied to the coordinates of individual trees to determine the position and orientation of each corresponding 50 × 50 m plot (0.25 ha). Then, zonal statistics were used to get the mean and median of satellite-obtained canopy height (Lang et al. 2023) and normalized difference vegetation index (NDVI) of the data collection period (Planet Labs PBC 2022) for each plot. In R (R Core Team 2023), correlations were tested between AGB_m_ and satellite-obtained canopy height and NDVI. AGB_m_ correlated more strongly with median canopy height (*r* = *0.795, p* < *0.001*) followed by mean canopy height (*r* = *0.794, p* < *0.001*) and mean NDVI (*r* = *0.481, p* < *0.001*). This suggests that the median better reflects typical canopy structure by reducing the influence of rare emergent trees that may inflate the mean canopy height.

Following the approach used in Sect. 2.2.1 to examine relationships between AGB and ecological determinants, we used linear mixed-effects models to analyze the relationship between AGB_m_ and canopy height. Again, we tested four variations (fixed intercept and fixed slope, fixed intercept and varying slope, varying intercept and fixed slope, varying intercept and varying slope) of single-factor linear mixed-effects models with forests treated as the random factor (Kuznetsova et al. 2017). The model with the lowest Akaike information criterion was retained (Bozdogan 1987) and model fit was assessed by Restricted Maximum Likelihood (REML). The retained model had a varying intercept and a fixed slope and allowed to extrapolate AGB_m_ beyond the sampled plots (Eq. 2):

2 $${AGB}_m=33.2071\ast\exp\left(0.0504\ast{Height}_L\right)$$

where AGB_m_ = aboveground biomass (Mg/ha) obtained from manual forest inventory and Height_L_ = median of canopy height (m) per plot obtained from Lang et al. (2023, *t* = *5.1, p* < *0.001*). Based on the relationship between plot-AGB_m_ and satellite-obtained canopy Height_L_, we could extrapolate AGB from the plots to the whole forest patch using the raster calculator in QGIS (QGIS Development Team 2023). The spatial resolution of the plots (50 × 50 m) was maintained, and the aligned raster data could later be used to generate difference maps with the AGB map by Harris et al. (2021, *r* = *0.6, p* < *0.001*).

In QGIS (QGIS Development Team 2023), we used zonal statistics to sum AGB and calculate its mean and standard deviation per forest. These values were then fed into the error propagation calculation for converting AGB to carbon (Eq. 3, Goodman 1960):

3 $${\sigma }_{AGB*Carbon}= \sqrt{\left({\upsigma }_{AGB}^{2}+{\upmu }_{AGB}^{2}\right)\left({\upsigma }_{Carbon}^{2}+{\upmu }_{Carbon}^{2}\right)-({\upmu }_{AGB}^{2}*{\upmu }_{Carbon}^{2})}$$

where ${\sigma }_{AGB*Carbon}$ is the standard deviation of the product of our estimated AGB and the carbon conversion factor as quantified by Martin et al. (2018), ${\upsigma }_{AGB}^{2}$ is the variance of AGB, ${\upmu }_{AGB}^{2}$ is the squared mean of AGB, ${\upsigma }_{Carbon}^{2}$ is the variance of carbon (0.002), and ${\upmu }_{Carbon}^{2}$ is the squared mean of carbon (0.456) (Martin et al. 2018). Total AGB uncertainty per forest was calculated as (Eq. 4, adapted from Taylor (1997)):

4 $${\sigma }_{sumAGBforest}={\sigma }_{AGBPerPixel}*\sqrt{\frac{n}{1+2*r(d)}}$$

where ${\sigma }_{sumAGBforest}$ is the uncertainty of the total AGB per forest, σ_AGBPerPixel_ is the uncertainty of AGB per 50 × 50 m pixel, *n* represents the number of pixels per forest, and *r(d)* is the correlation coefficient of a pixel with its eight immediate neighboring pixels corresponding to a 50 m radius. The distance of 50 m was chosen during field data collection and applied in the analysis to avoid biases due to spatial autocorrelation. Finally, we validated the extrapolated AGB by comparing it with input plot data using a Wilcoxon test (Bauer 1972; R Core Team 2023). We compiled published AGB data from the same or nearby forests to compare isolated patches with larger, differently managed, and differently estimated forest tracts.

#### Terrestrial laser scanning (TLS) data

TLS-data from 86 subplots were processed and co-registered in SCENE (version 2023.1.0, FARO Technologies Inc., 2023). We generated 50 point-clouds, each consisting of 167 million points on average, with a mean point error of 22 mm. In 36 subplots, scan registration failed due to very dense understory vegetation, hindering automatic merging of adjacent scans. In seven subplots, we did not identify tree species and therefore had no corresponding wood densities, leading to 43 completely analyzed subplots.

The point-clouds were processed in the Forest Structural Complexity Tool (FSCT), which is sensor-agnostic and known for high accuracy (Krisanski et al. 2021; Boroujeni et al. 2024). We segmented the point-clouds automatically into ground, leaf, and stem points, isolated individual trees, and fitted cylinders for tree volume estimation (Krisanski et al. 2021; Appendix Fig. 8). Visual inspection of the segmented point-clouds was performed in CloudCompare (Girardeau-Montaut 2023).

**Fig. 3:**
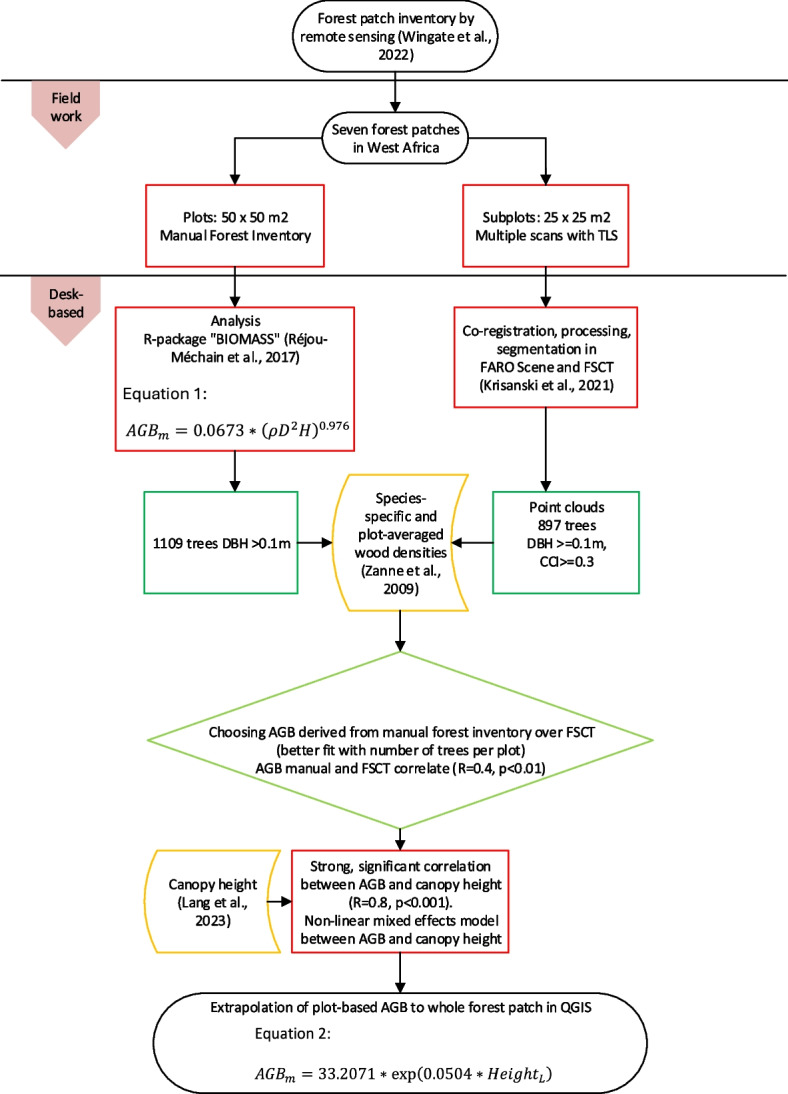
Workflow illustrating the selection of forest patches, establishment of plots for data collection, and subsequent analysis comparing aboveground biomass (AGB) obtained by manual inventory and TLS

The output of FSCT was filtered by DBH $\ge$ 10 cm and circumference completeness index (CCI) $\ge$ 0.3 (Krisanski et al. 2021), to align with the manual inventory and reduce noise (Fig. 3). The CCI measures the completeness of a scanned circular object, such as a stem or branch. Apparent stems scanned only from one side were excluded as noise. These filters were confirmed as best fit by an analysis of Euclidean distance between the manual inventory data and FSCT output, and on average, 60% of the originally detected ‘trees’ were filtered out this way. Wood density for each FSCT subplot was derived from the manual inventory by assigning species-specific wood densities and calculating a basal-area–weighted mean. Basal area was computed for each tree as $\uppi *{(\frac{\mathrm{DBH}}{2})}^{2}$ (Bettinger et al. 2017), and subplot-level mean wood density was obtained by weighting species wood densities by their relative basal area contributions. This mean wood density was then applied to the TLS-derived stem volumes to convert them into subplot-level aboveground biomass (AGB_TLS_). Correlations were used to compare forest characteristics estimated by manual inventory and TLS. Analysis of variance (ANOVA) was used to detect peculiarities across forest patches.

Based on five scans per plot, we calculated the stand structural complexity index (SSCI, Ehbrecht et al. 2017; Hepner et al. 2025a). This index constructs polygons of open space around the scanner position, connecting points where plant matter reflected the laser beams. SSCI is defined as (Eq. 5):

5 $${SSCI= MeanFrac}^{\mathrm{ln}(ENL)}$$

where *MeanFrac* refers to the mean of the fractal dimension index of 1280 polygons surrounding the scanner, derived from the perimeter and area of these polygons. ENL refers to the effective number of layers, quantifying 20 cm voxels filled with plant material in 1 m layers from the scanner to the canopy top (Ehbrecht et al. 2017). SSCI is a powerful metric for quantifying three-dimensional forest structure, and higher structural complexity is typically associated with greater primary productivity, improved microclimate regulation, higher faunal diversity, and increased habitat availability (Coverdale and Davies 2023).

All plot- and subplot-level data generated in this study (AGB) are archived and openly available (Hepner et al. 2026). Related metrics on SSCI and tree species composition are archived under Hepner et al. (2025b) and Ifejika Speranza and Agonvonon (2024), respectively.

## Results

### AGB and carbon in the seven studied forest patches

AGB_m_ ranged from 85 Mg/ha in Ikot to 199 Mg/ha in Ngam-Kondomeyos, corresponding to 39 Mg/ha and 91 Mg/ha carbon (Table 1). The smallest forest Koui (18 ha), stored 351 Mg AGB, and the largest forest Iko (1163 ha), stored 44,812 Mg AGB.

**Table 1:** Characteristics of the studied forests, including area, aboveground biomass (AGB) per hectare, and carbon content. Means are shown with standard deviations in brackets. AGB per hectare varies with forest type (lower in swamp forests, e.g., Ikot), forest integrity (lower in degraded forests, e.g., Ewè-Adakplamè), and generally increases toward the equator. Mean AGB in the sampled plots was often higher than the mean AGB of the entire forest, because areas with tree cover < 10% were not sampled

Forest	Area (ha)	Mean AGB per plot (Mg/ha)	Mean AGB per forest (Mg/ha)	Mean carbon per forest (Mg/ha)
Koui	18	153 (± 98)	116 (± 42)	53 (± 19)
Ewè-Adakplamè	218	116 (± 48)	104 (± 17)	44 (± 8)
Hlanzoun	676	131 (± 58)	108 (± 31)	52 (± 14)
Iko	1163	309 (± 106)	188 (± 41)	86 (± 19)
Ikot	1116	46 (± 28)	85 (± 23)	39 (± 10)
Mbangassina	145	293 (± 134)	259 (± 43)	118 (± 20)
Ngam-Kondomeyos	399	301 (± 69)	207 (± 28)	94 (± 13)

#### Spatial patterns of AGB within forests

**Fig. 4:**
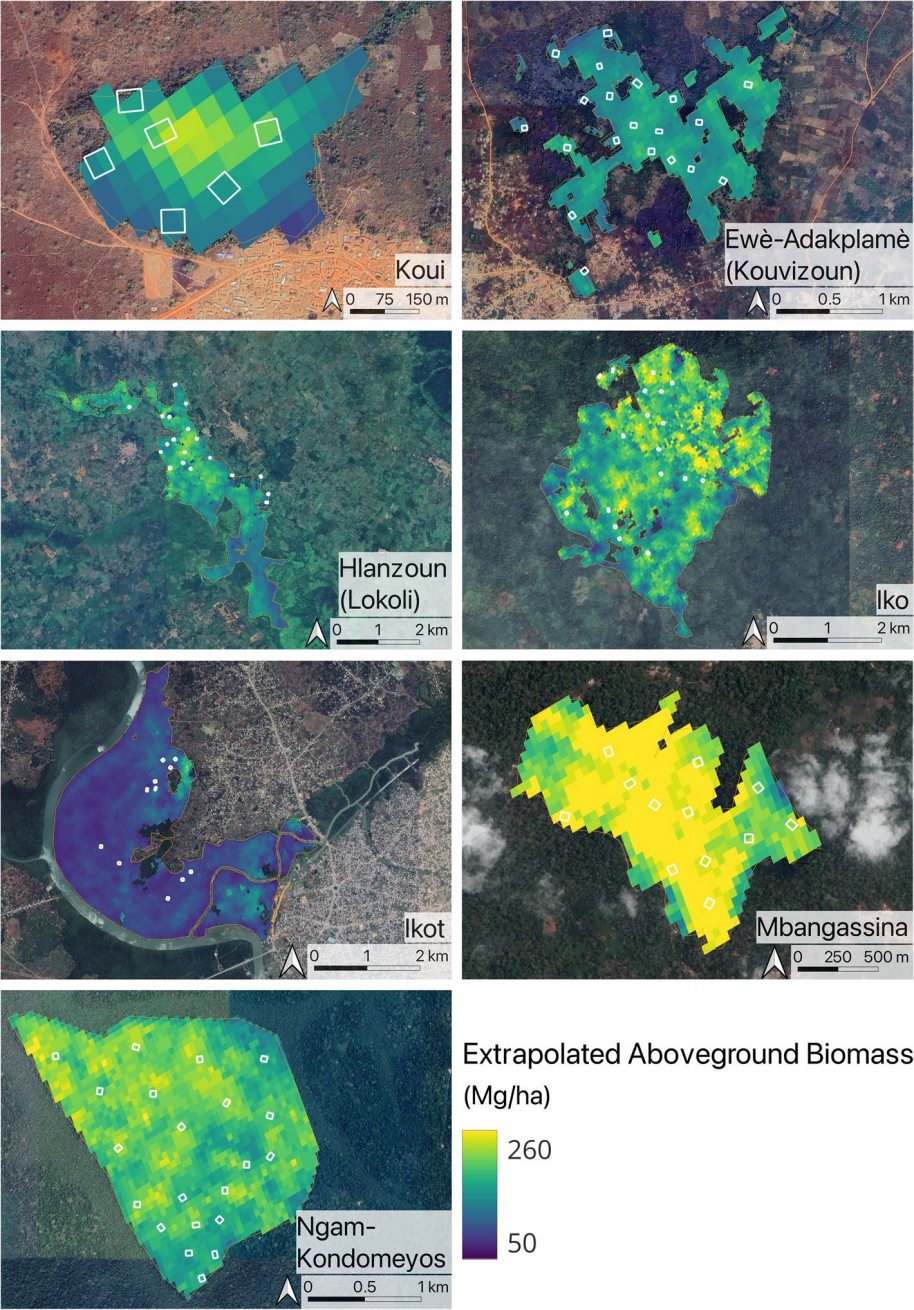
Maps of the forest patches showing the spatial distribution of aboveground biomass (AGB; Mg/ha) and forest inventory plots (white). In Koui, AGB was higher in the interior, likely due to increased water availability in a topographic depression. In Ewè-Adakplamè, the forest was strongly fragmented, with high AGB persisting only in the former interior. In Hlanzoun, AGB increased toward the interior (*r* = 0.1, *p* < 0.001), but water-saturated areas in the south reduced AGB near both the interior and edges. In Ikot, AGB increased slightly toward the interior (*r* = 0.2, *p* < 0.001), although proximity to the periodically rising Kwa Ibo River appeared to reduce AGB locally. In Iko (*r* = 0.06, *p* < 0.05), Mbangassina, and Ngam-Kondomeyos, AGB was generally homogeneous, with some local decreases in previously logged areas. Background imagery from Google Maps

A linear mixed-effects model with log-transformed AGB, distance from the forest edge as a fixed effect, and forest as a random intercept *(lmer(log(AGB)* ~ *distance* + *(1 | Forest)))*, showed that AGB_m_ increased toward forest interiors (*t* = *2, p* < *0.005*). Across forests, distances from plot to forest edge and AGB_m_ differed significantly (*ANOVA, p* < *0.001,* Fig. 4)*.* At the forest level, Hlanzoun (*r* = 0.2, *p* < 0.001), Iko (*r* = 0.06, *p* < 0.05), and Ikot (*r* = 0.2, *p* < 0.001) showed increasing AGB_m_ toward the forest interiors.

Separate linear mixed-effects models with distance from the forest edge as a fixed effect and forest as a random intercept suggested that diameter (*t* = *3, p* < *0.005*), tree height (*t* = *3, p* < *0.005*), and wood density (*t* = *4, p* < *0.001*) increased toward the forest interior. Tested individually, the forests of Hlanzoun and Ikot showed the same edge effects. For wood density alone, the relationship was significant in Koui (*r* = *0.13, p* < *0.01*), Iko (*r* = *0.08, p* < *0.001*), and Mbangassina (*r* = *0.07, p* < *0.05*).

Wilcoxon test comparing field-estimated and extrapolated AGB per plot indicated that the extrapolation from plot to forest using satellite-obtained canopy height was accurate for Koui, Ewè-Adakplamè, and Hlanzoun. However, it slightly overestimated AGB in Iko and Ngam-Kondomeyos, while underestimating AGB in Ikot.

### Ecological determinants of AGB

AGB_m_ is composed of wood volume and wood density. Accordingly, we found strong correlations between AGB_m_ and both basal area (*t* = *6, p* < *0.001*) and tree height (*t* = *4.8, p* < *0.001*). However, no correlation was found between AGB_m_ and wood density.

AGB_m_ and tree species richness were both low in the swamp forests (Ikot, Hlanzoun) and the moist semi-deciduous forests (Koui, Ewè-Adakplamè) and high in the moist forests toward the equator (Iko, Mbangassina, Ngam-Kondomeyos). AGB_m_ and tree species richness did not correlate (Appendix Fig. 9). AGB_m_ correlated well with the number of tree stems (DBH > 10 cm, *t* = *2.8, p* < *0.01*), the standard deviation of tree height (*t* = *7.36, p* < *0.001*), but neither with stand structural complexity (SSCI) nor with canopy openness. Interestingly, wood density was higher in shorter trees (*t* = *−2.3, p* < *0.05*), but no correlation was found with the number of trees, tree height variability, SSCI, or canopy openness.

### Comparisons of AGB across regions and methods

#### Regional AGB comparison

We compared our AGB estimations of isolated forest patches with published AGB data from forests in the same region. Our AGB estimations were, in most cases, lower than comparable forest sites in the same regions (Table 2).

**Table 2:** Aboveground biomass (AGB) per hectare estimated in this study using manual inventory, compared to values from nearby forests. Overall, AGB in our study plots is slightly lower than reported for adjacent forest patches

Forest patch	AGB_m_ this study (Mg/ha)	AGB comparison value (Mg/ha)	Description of comparison forest and distance to study forest patches (km)	Forest size (ha)	Reference
Koui	116	209	Closed canopy forest Fazao Malfakassa National Park, 13 km	192,000	(Atsri et al. 2020)
		129	Dense forest in same ecological zone	604,000	(Dangbo et al. 2020)
		104	Sabi sacred forest, 100 km	240	(Lynch et al. 2018)
		131	Kala sacred forest, 100 km	500	(Lynch et al. 2018)
Ewè-Adakplamè	104	829	Lama forest reserve, 75 km	4780	(Biah et al. 2024)
Hlanzoun	108	488	Intact parts of same Hlanzoun forest (also known as Lokoli swamp forest)		(Biah et al. 2024)
		199	Disturbed parts of same Hlanzoun forest (also known as Lokoli swamp forest)		(Biah et al. 2024)
Iko	188	223	Intact forests with little or no human disturbances in Cross River State	729,000	(Amuyou et al. 2022)
		107	Disturbed forests with signs of logging, fire, agriculture in Cross River State	729,000	(Amuyou et al. 2022)
Mbangassina	259	421	Belabo Sub-Divion, Lom & Djeren forest management unit, 200 km	4,590	(Chimi et al. 2018)
Ngam-Kondomeyos	207	401	Dja Biosphere Reserve, 100 km	526,000	(Djuikouo et al. 2010)

#### Comparing AGB from manual inventory and TLS

We compared manual tree inventory and TLS-data from 43 subplots (25 × 25 m). There were several moderate correlations between forest characteristics estimated by manual inventory and TLS, such as the number of detected trees, tree height, and AGB (Table 3). Manual inventory detected more trees and resulted in higher DBH and AGB and lower tree heights than TLS.

**Table 3:** Mean values and standard deviations of key forest parameters, compared between manual forest inventory and TLS measurements per subplot. Correlations between the two methods are generally moderate. Significance of correlations is indicated by asterisks (*: *p* ≤ 0.05; **: *p* ≤ 0.01; ***: *p* ≤ 0.001)

Mean (and standard deviation) in subplots	Manual forest inventory (n trees = 1,109)	TLS (n trees = 897)	Spearman correlation
Number of trees	26 (± 10)	21 (± 13)	0.4 **
DBH (cm)	26 (± 6)	20 (± 5)	0.4 *
Max DBH (cm)	72 (± 30)	44 (± 23)	0.1
Tree height (m)	16 (± 1)	19 (± 7)	0.5 ***
Max tree height (m)	25 (± 4)	29 (± 11)	0.7 ***
AGB (Mg)	15 (± 11)	9 (± 5)	0.4 *

**Fig. 5:**
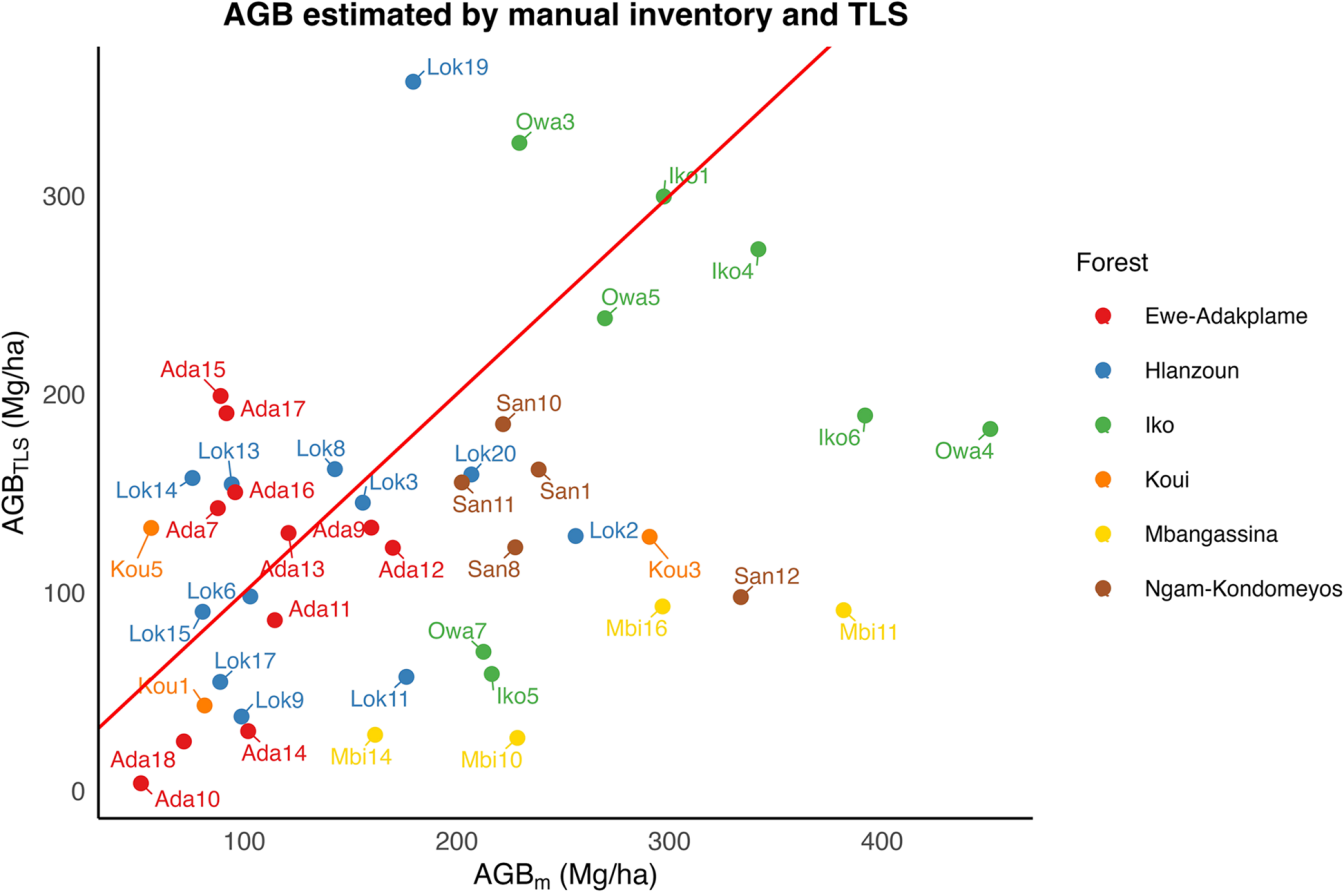
Comparison of aboveground biomass (AGB) per plot (*n* = 43) estimated by manual inventory using allometric equations (x-axis, AGB_m_) and by terrestrial laser scanner (TLS) with Forest Structural Complexity Tool (FSCT, Krisanski et al. 2021, y-axis, AGB_TLS_). The red line indicates where x and y values are equal. Points are colored by forest: Ada = Ewè-Adakplamè, Eke = Ikot, Iko and Owa = Iko, Kou = Koui, Mbi = Mbangassina, Lok = Hlanzoun, San = Ngam-Kondomeyos

AGB as estimated from manual inventory and from TLS correlated moderately (*r* = *0.4, p* < *0.01,* Table 1*).* Manual inventory resulted in higher estimates of AGB than did TLS in 30 of 43 plots (Fig. 5). Differences between AGB_m_ and AGB_TLS_ ranged from −93% to + 136%. According to an ANOVA, the discrepancies between AGB_m_ and AGB_TLS_, as well as the amount of noise in TLS point-clouds were evenly distributed across the forests.

## Discussion

### AGB and carbon in seven forest patches

We confirmed the hypothesis that the magnitude of AGB and carbon vary across the forest patches, indicating environmental and disturbance gradients. AGB in the sampled forests was higher in the biome of moist broadleaf forests compared to forests in savanna, grasslands, and shrublands. This pattern aligns with global gradients of precipitation and water availability, which, together with soil fertility, elevation, and disturbance, are major drivers of AGB (Chave et al. 2019), generally leading to higher AGB toward the equator (Lewis et al. 2013). Soil water saturation in swamp forests (Hlanzoun, Ikot) limited AGB and tree species richness (see also Koponen et al. 2004; Rodríguez-González et al. 2010). Whereas, anthropogenic degradation processes such as frequent logging constrained biomass accumulation in Ewè-Adakplamè and Ikot (Hepner et al. 2025a).

#### Spatial patterns of AGB within forests

We confirmed the hypothesis that AGB varies within the different forest patches, reflecting disturbance gradients. In fact, extrapolation of our plot-based measurements revealed edge effects in AGB. These patterns are consistent with previous studies (Chaplin-Kramer et al. 2015; Laurance et al. 1997; Mo et al. 2023; Ordway and Asner 2020) and are especially evident in isolated forest patches such as Koui, Ewè-Adakplamè, Hlanzoun, and Ikot where surrounding tree cover within 1 km is sparse (connectivity < 30%, Hepner et al. 2025a). Likely mechanisms include altered microclimate characterized by stronger winds, higher temperatures, and increased risk of desiccation, which lead to changes in species composition and forest structure close to edges (Chaplin-Kramer et al. 2015; Laurance et al. 1997). In addition, a high prevalence of anthropogenic fires further reduces AGB close to edges (Chaplin-Kramer et al. 2015), as observed in Koui, Ewè-Adakplamè, and Iko (Chuvieco et al. 2018). These effects can affect tree architecture, which additionally decreases AGB close to forest edges (Nunes et al. 2023).

Edge effects in tree diameter, tree height, and wood density were not visible in Ewè-Adakplamè and Ngam-Kondomeyos. Ewè-Adakplamè is likely too fragmented to show clear edge gradients, whereas Ngam-Kondomeyos shows high landscape connectivity to surrounding woody vegetation (90% tree cover within a 1 km buffer; Hepner et al. 2025a), allowing adjacent trees to buffer edge effects. In four of the seven forests, wood density was lower in trees close to edges. Edges promote fast-growing, light-demanding pioneers which invest less resources in wood robustness and density and are therefore lighter-wooded (Ghazoul and Sheil 2010; Nunes et al. 2023). Trees exposed to a newly formed edge can also adapt wood structure and density to reduce desiccation risk (Silva Da Costa et al. 2020). In the swamp forest of Hlanzoun, growth rate in the forest interior was likely limited by chronic soil water saturation from the Hlan-river (Rodríguez-González et al. 2010), leading to higher wood densities. More generally, flooding strongly constrains tree growth and survival of most species. However, comparable data from Western-African floodplains are lacking (Yamanoshita et al. 2001; Koponen et al. 2004; Parolin and Wittmann 2010; Smith et al. 2022).

### Ecological determinants of aboveground biomass

We confirmed the hypothesized correlation between AGB and (i) basal area, and (ii) tree height. In contrast, despite wood density being a fundamental factor for AGB, our data revealed no direct correlation with biomass. Wood density and tree volume appear largely uncorrelated and control AGB independently (Phillips et al. 2019), meaning that high wood density can compensate for low wood volume and vice versa. At the individual tree scale, we observed that wood density was higher in shorter trees. Ecologically, high wood density is typical of slow-growing, shade-tolerant species, which invest more in structurally robust stems and tend to be shorter than fast-growing heliophyte species (Ghazoul and Sheil 2010; Mo et al. 2024). Recent analyses in temperate European forests suggest that tree dimensions can even explain part of intraspecific wood density variation (Cuny et al. 2025). It is important to note that carbon concentrations are negatively related to wood density (Martin et al. 2018). Additional empirical data on wood density and corresponding carbon concentrations are therefore required to improve our understanding of AGB-determinants in the species-rich forests of the tropics (Martin et al. 2018; Mo et al. 2024).

In our 0.25 ha plots, AGB did not correlate with tree species richness. This finding is consistent with other studies at similar plot sizes and in comparable environments (Ali et al. 2016; ForestPlots.net et al. 2021; Cuni-Sanchez et al. 2021). However, Sullivan et al. (2017) and Dyola et al. (2022) found such correlations in smaller plots of 0.04 ha. While niche complementarity theory suggests that higher species richness enhances biomass through resource-use efficiency (Ali et al. 2019a), factors such as historical disturbances (Mitchard 2018) and ongoing climate stressors may obscure the relationship between AGB and tree species richness (Yang et al. 2024).

The relationship between AGB and forest structure is not entirely clear. We found correlations between AGB and the number of trees, tree heights, and height variability, but not with stand structural complexity as defined by Ehbrecht et al. (2017). While it seems intuitive that more tree stems would correlate with higher AGB, this is not necessarily the case (Lewis et al. 2013) as a few large trees can offset the AGB of many small ones (Ali et al. 2019b). Lang et al. (2023) confirmed a correlation between AGB and tree height. Structurally complex forests are known to capture light more efficiently, pack the canopy more densely, and store more carbon (Coverdale and Davies 2023). Ali et al. (2019b) identified stand structural complexity, based on DBH and tree height variance, as a key biotic factor influencing AGB, with tree species richness contributing to AGB through greater size variability and complexity. However, Ehbrecht et al. (2021) found no correlation between SSCI and basal area, a proxy for AGB, highlighting that the bidirectional relationship between forest structural complexity and AGB requires further research (Coverdale and Davies 2023).

Regarding wood density on a plot scale, we did not find relationships with the number of trees per plot, tree height variability, stand structural complexity (SSCI), or canopy openness. Wood density might be more influenced by tree genetics and local edaphic conditions than by forest structure (Phillips et al. 2019).

### Comparison of AGB across regions and methods

#### Regional AGB comparison

Confirming our hypothesis, AGB is higher in formally protected and often larger forests as compared to the unprotected small forest patches, exposed to various edge effects. These differences can have ecological, as well as methodological reasons. It is likely that edge effects, low landscape connectivity, and anthropogenic disturbances constrain the accumulation of AGB in small forest patches (Laurance et al. 1997). In degraded forests, such as Ewè-Adakplamè and Ikot (Houngnon et al. 2021; Hepner et al. 2025a), logging was prevalent, and AGB below its potential.

However, the considerable discrepancies between our AGB-estimates and those reported by Biah et al. (2024) for the Hlanzoun forest likely arise primarily from methodological differences. Biah et al. (2024) reported AGB values two to four times higher than ours but identified only eight dominant species, whereas we distinguished 30 species, allowing for species-specific wood density and allometric variation. Taxonomic aggregation can inflate biomass estimates if dominant species with high wood density or large stature are assumed to represent the entire stand. In addition, Biah et al. (2024) applied a generic biomass expansion factor following the Intergovernmental Panel on Climate Change (IPCC, 2006), whereas swamp forests exhibit distinct structural adaptations to chronic waterlogging, including multi-stemming and a lower proportion of large-diameter stems (Rodríguez-González et al. 2010; Lewis et al. 2013). Applying generic expansion factors developed largely for *terra firme* forests may therefore lead to systematic overestimation of AGB in swamp ecosystems. Also, while our estimates resolved spatial heterogeneity at 50 × 50 m, Biah et al. (2024) assumed homogeneous AGB across areas exceeding 550 ha, which likely smooths fine-scale variation and can further contribute to inflated mean AGB values. Finally, Biah et al. (2024) measured all trees with DBH ≥ 5 cm in contrast to the threshold of ≥ 10 cm used in this study. However, this difference is unlikely to explain the higher AGB estimates. Atsri et al. (2020) showed that including trees between 5 and 10 cm DBH does not significantly affect AGB estimates in Togolese forests. Small trees contribute only marginally to biomass (i.e., 10% in forests with AGB > 175 Mg/ha;Schroeder et al. 1997; Duncanson et al. 2021), and the largest 1% of trees account for more AGB than the remaining 99% combined (Ali et al. 2019b). Taken together, the methodological differences between Biah et al. (2024) and this study provide a plausible explanation for the substantial discrepancies in AGB-estimates for the Hlanzoun forest.

**Fig. 6:**
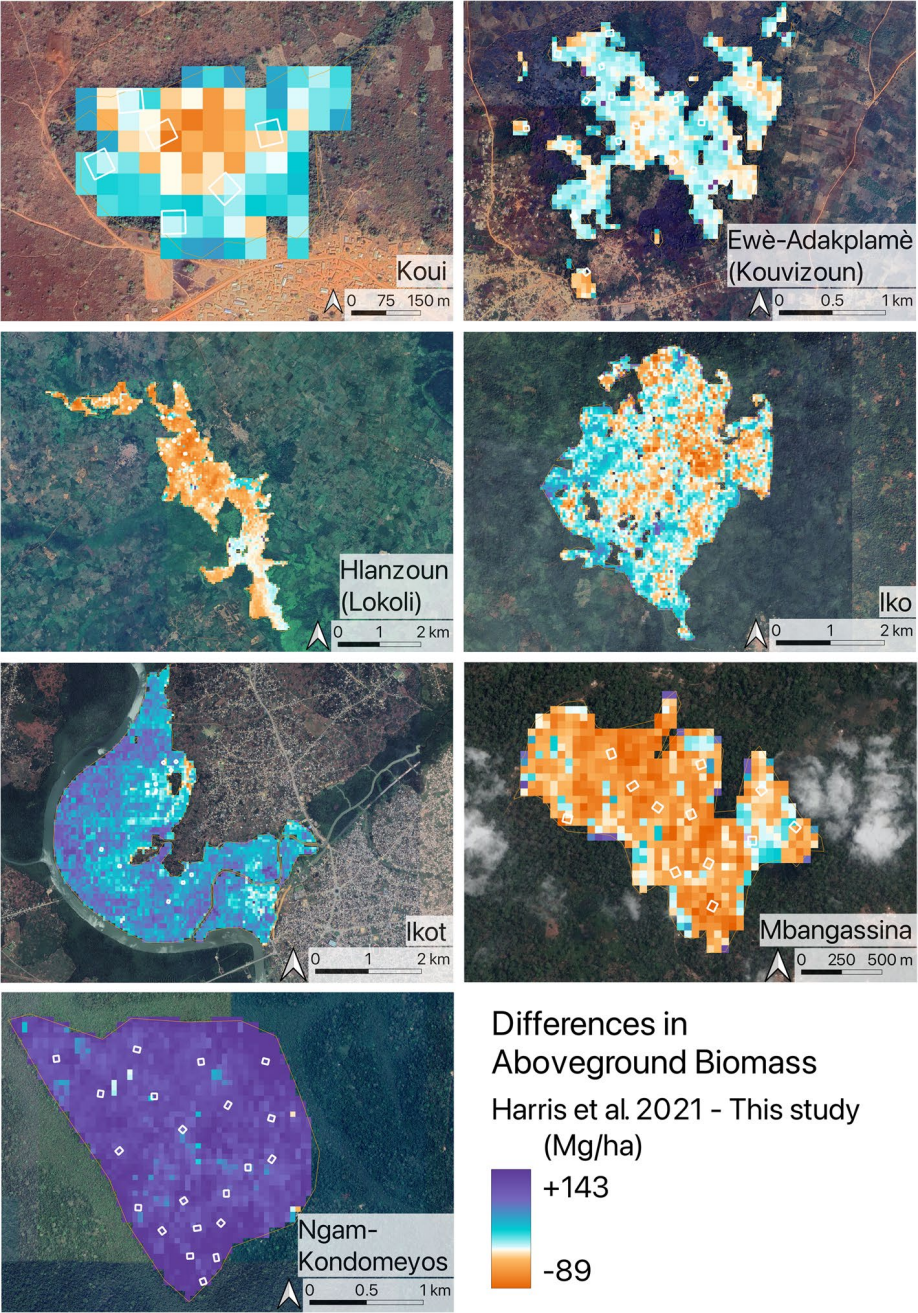
Difference maps of aboveground biomass (AGB; Mg/ha) between Harris et al. (2021) and this study across seven Western-African forests. Blue indicates higher estimates, and orange indicates lower estimates by Harris et al. (2021) compared to this study. White squares mark the locations of plots. Differences are generally low and homogeneous in Koui, Ewè-Adakplamè, and Iko, but larger differences occur in swamp forests (Hlanzoun and Ikot) and in forests embedded in agroforestry or wetlands (Mbangassina, Ngam-Kondomeyos). Background imagery from Google Maps

The AGB-map by Harris et al. (2021) showed generally good agreement with our field-based estimates, supporting the validity of global AGB products at broad scales. However, substantial local deviations (–89 to + 143 Mg/ha; Fig. 6) occurred in the swamp forests (Hlanzoun, Ikot) and in forests embedded in agroforestry and woody wetlands (Mbangassina, Ngam-Kondomeyos). In swamp forests, distinct species composition and tree architecture shaped by hydrological conditions may diverge from assumptions underlying *terra firme*–based AGB extrapolation (Rodríguez-González et al. 2010; Lewis et al. 2013). In forests embedded in agroforestry systems or densely vegetated wetlands, canopy structures and spectral signals can be similar to surrounding land covers, making it difficult to distinguish them with spaceborne observations alone. Moreover, because forest AGB is dynamic and varies interannually due to growth, disturbance, and recovery processes (Chave et al. 2019; Harris et al. 2021), some degree of deviation is expected when comparing temporally mismatched datasets. Local discrepancies reflect ecosystem-specific limitations of global AGB extrapolation.

#### Comparing AGB from manual inventory and TLS

AGB obtained by manual inventory correlated with AGB derived from TLS. However, manual inventory showed practical advantages: it was faster (12 vs. 16 person days/ha for TLS) and successful in all plots, whereas registration and segmentation of TLS data succeeded in only 60% of scanned plots. Dense and structurally complex tropical forests remain challenging for TLS due to occlusion, segmentation difficulties, and limited objective methods to evaluate point-cloud quality and accuracy for tree volume reconstruction (Momo et al. 2018; Martin-Ducup et al. 2021; Demol et al. 2022). In some cases, leaves, lianas, and epiphytes obscured stems, preventing detection by TLS unless vegetation was cleared (see Burt et al. 2018).

Our data showed no evidence that agreement between AGB estimates from manual inventory (AGB_m_) and TLS (AGB_TLS_) improved in more open or structurally simple forests, or for taller trees. For both methods, AGB estimation became more uncertain with increasing forest density, tree height, and total AGB (Appendix Fig. 7). While Demol et al. (2022) reported better TLS performance in tall trees, Momo Takoudjou et al. (2018) warned about increased occlusion caused by complex tree architecture and crown overlap in large trees.

Although progress is being made in species identification from point clouds (Åkerblom et al. 2017; Puliti et al. 2025), these approaches are not yet reliable in tropical forests. Consequently, TLS does not replace manual inventory, as species identification remains necessary to assign wood density, which can vary substantially among species.

Based on the number of trees detected (Table 3), manual inventory was selected over TLS as the reference for AGB estimation. Owing to the absence of species-specific equations for the region (GlobAllomeTree 2024) and the inferior performance of region-specific models (Feldpausch et al. 2012) compared with the pantropical equation of Chave et al. (2014), we applied the latter. While this approach simplifies reality by neglecting morphological plasticity (Calders et al. 2022a, b), it also introduces uncertainty due to limited calibration data and representativity (Demol et al. 2022). Allometric equations are known to perform poorly in dense, complex forests and for large trees (Gonzalez de Tanago et al. 2018; Disney et al. 2018; Burt et al. 2018; Calders et al. 2022a, b). Although we found no correlation between tree form (DBH, height) and plot-level structural variables, tree allometry is still likely influenced by stand structure and environmental conditions that cannot be captured by a single equation (Loubota Panzou et al. 2021; Sullivan et al. 2017). Developing individual- or species-specific allometric equations from the TLS point-clouds was beyond the scope of this study and impractical due to poor automatic tree isolation in FSCT (Appendix Fig. 8b).

Direct AGB measurement through tree felling remains the most accurate method (Clark and Kellner 2012) but is not feasible in sacred and vulnerable forest patches. Combining complementary non-destructive approaches therefore provides a pragmatic compromise, particularly in data-scarce regions.

### Limitations

This study quantified forest AGB and carbon based on a single field campaign. Given that forest AGB is dynamic and can change rapidly over short timescales (Hansen et al. 2020; Binkley 2021), our estimates represent a temporal snapshot (Hansen et al. 2020). Canopy height data preceded our field measurements by two to three years, potentially contributing to mismatches due to forest growth, disturbance, or recovery dynamics. In addition, extrapolating plot-based AGB using satellite-derived canopy height may be affected by plot-edge effects (i.e., trunk of a tree is in plot but crown is mainly outside of plot) and GPS inaccuracies (± 3 m), with errors propagating across spatial scales (Réjou-Méchain et al. 2019). Further uncertainty arises from limited regional data on wood density and carbon concentration, as well as a lack of high-resolution spatial data on soil properties and forest management history, both of which can influence AGB (Lindsell and Klop 2013; Traoré et al. 2024).

### Broader implications

This study provides plot- and tree-level estimates of AGB and species composition from small tropical forest patches in an understudied region of Western-Africa. Such fine-scale field data remain scarce, particularly for fragmented landscapes and swamp forests, yet are essential for calibrating and validating regional and global biomass products (Chave et al. 2019). By providing both manual inventory and TLS data, this study contributes empirical benchmarks for improving biomass estimation in complex tropical forest systems and supports more informed interpretation of remotely sensed AGB products used in ecological and climate-related assessments.

### Outlook

Future research should focus on: i) improving regional coverage of species-specific wood density and carbon concentration data for tropical trees (Réjou-Méchain et al. 2019); ii) advancing laser scanning hardware to increase point-cloud quality in structurally complex forests (Abegg et al. 2017); iii) developing more robust tree segmentation and isolation algorithms to reduce dependence on static allometric equations and enable structure-based biomass estimation (Calders et al. 2022a, b); iv) advancing acquisition strategies by a) integrating terrestrial and aerial laser scanning to improve crown representation in biomass estimation (Zhou et al. 2023) and b) conducting canopy-based laser scanning in selected individual trees to quantify uncertainty related to crown occlusion and benchmark TLS-derived biomass estimates on a plot scale (D’hont et al. 2025), and finally v) expanding pool-wise carbon assessments beyond aboveground biomass to include belowground, soil, microbial, fungal, and faunal components (Ashton et al. 2012). Continued field-based studies will remain essential for calibrating and validating satellite-derived biomass products, which are increasingly important for large-scale assessments of forest carbon stocks (Calders et al. 2022a, b; European Space Agency 2025).

## Conclusion

Small and isolated forest patches in Western-Africa store substantial amounts of aboveground biomass, but consistently less than larger and more connected forest systems. Our results show that AGB, tree height, and wood density increase toward forest interiors, confirming strong edge effects that are particularly pronounced in fragmented patches. Swamp forests further diverge from *terra firme* forests in structure, species composition, and biomass accumulation, underscoring the need to treat these ecosystems separately in regional and global AGB assessments.

Manual forest inventory and terrestrial laser scanning produced correlated AGB estimates, but TLS remains challenged in dense and structurally complex tropical forests due to occlusion, segmentation failures, and the inability to reliably identify tree species. Under current conditions, manual inventory remains a more robust approach for estimating AGB in small tropical forest patches, while TLS provides complementary structural information.

Overall, our findings demonstrated that small forest remnants are shaped by fragmentation, edge effects, and local environmental constraints. Explicitly incorporating these patch-specific dynamics is essential for improving biomass estimates and for understanding the role of fragmented tropical forests in regional carbon budgets.

## Figures and Tables

**Fig. 7:**
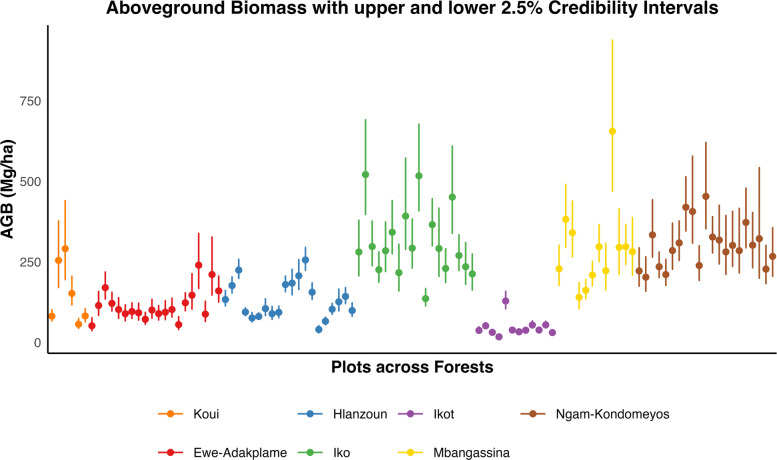
Aboveground biomass (AGB) per plot across forests spanning the Guinea Savanna (Togo: Koui; Benin: Ewè-Adakplamè and Hlanzoun) to the Guineo-Congolian zone (Nigeria: Iko and Ikot; Cameroon: Mbangassina and Ngam-Kondomeyos). Estimation uncertainty increases with AGB, as quantified using a Monte-Carlo approach. Swamp forests (Hlanzoun and Ikot) and disturbed forests (Ewè-Adakplamè and Ikot) exhibit lower aboveground biomass

**Fig. 8:**
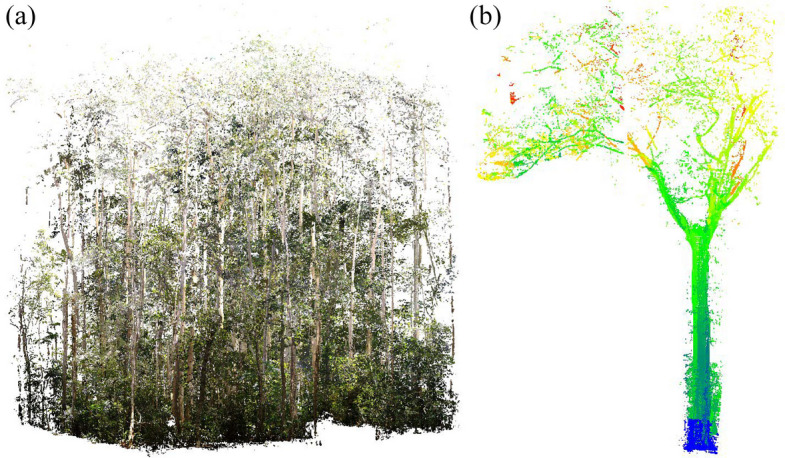
**a** Plot point clouds (25 × 25 m) were segmented into ground, leaf, and stem points using the Forest Structural Complexity Tool (FSCT, Krisanski et al. 2021). **b** Automatic tree isolation from the plot point cloud in FSCT is imperfect. In this example, points belonging to several trees (indicated by different colors) were incorrectly assigned to a single tree. Tree isolation is particularly challenging in areas with overlapping crowns

**Fig. 9:**
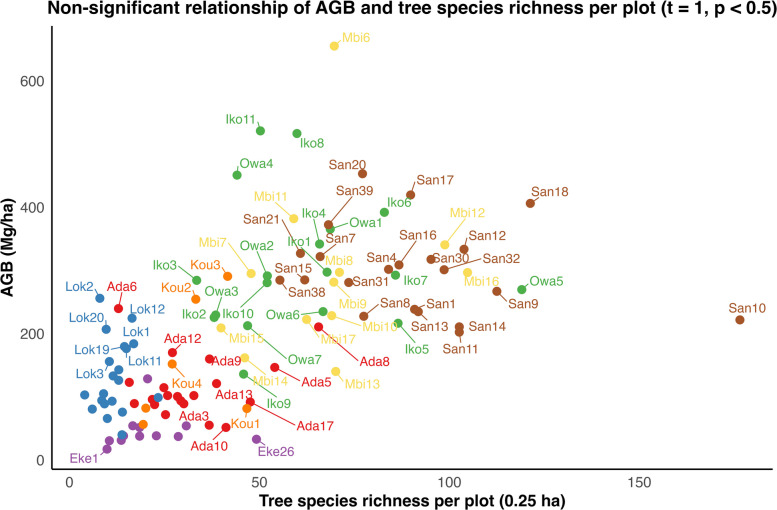
Linear mixed-effects model of tree species richness and aboveground biomass (AGB; Mg/ha) per 0.25-ha plot. Swamp forests exhibit lower tree species richness and AGB, and both variables tend to increase toward the equator. After accounting for forest identity as a random effect, there is no significant relationship between AGB and tree species richness. Forest abbreviations: Ada = Ewè-Adakplamè, Eke = Ikot, Iko and Owa = Iko, Kou = Koui, Mbi = Mbangassina, Lok = Hlanzoun, San = Ngam-Kondomeyos

## Data Availability

All plot- and subplot-level data generated in this study (AGB) are archived and openly available (Hepner et al. 2026). Related metrics on SSCI and tree species composition are archived under Hepner et al. (2025b) and Ifejika Speranza and Agonvonon (2024), respectively.

## Appendix

**Table 4:** Characteristics of the seven forest patches studied in Togo, Benin, Nigeria, and Cameroon. Column headers without cited sources refer to the authors’ own measurements and field observations. This table partly overlaps with that presented in Hepner et al. (2025a)

Nr.	Country	Forest name	Plots abbreviation	Coordinates (WGS 84, Latitude/Longitude)	Measured forest area (ha)	Number of plots	Vegetation type	Soil (International Union of Soil Sciences (IUSS) Working Group 2015)	Surrounding landcover
1	Togo	Koui	Kou	0° 43' 12"/8° 15' 36"	18	6	Moist semi-deciduous forest	Acrisol	Settlement/Agriculture/Savanna
2	Benin	Ewè-Adakplamè (also known as Kouvizoun sacred forest Adakplamè-Ewè)	Ada	2° 34' 12"/7° 28' 12"	218	20	Moist semi-deciduous forest	Acrisol/Lixisol	Settlement/Agriculture/Savanna
3		Hlanzoun (also known as Lokoli)	Lok	2° 15' 36"/7° 3' 36"	676	20	Swamp forest	Acrisol/Gleysol/Lixisol	Settlements/Agriculture/Wetlands
4	Nigeria	Iko	Iko & Owa	8° 15' 0"/5° 35' 24"	1163	18	Moist forest	Acrisol	Agriculture/Agroforestry
5		Ikot	Eke	7° 53' 24"/4° 39' 36"	1116	12	Swamp forest	Acrisol/Cambisol/Fluvisol	Settlement/Agriculture/Water
6	Cameroon	Mbangassina	Mbi	11° 35' 24"/4° 38' 24"	145	12	Moist forest	Ferralsol	Agriculture/Agroforestry
7		Ngam-Kondomeyos	San	11° 49' 48"/3° 2' 24"	399	21	Moist forest	Ferralsol	Wetlands/Agroforestry

Due to practical constraints during fieldwork, we also included the two forests, Iko and Ikot (Nigeria), although they slightly exceed the 1,000 ha threshold defined for small forest patches
